# Subcutaneous Dorsal Penile Vein Thrombosis or Penile Mondor's Disease: A Case Report and Literature Review

**DOI:** 10.1155/2019/1297048

**Published:** 2019-08-20

**Authors:** Adama Ouattara, Abdoul Karim Paré, Aristide F. Kaboré, Clotaire Yaméogo, Gnimdou Botcho, Désiré Ky, Abdoul Aziz Ouédraogo, Amidou Bako, Rigobert Kiba, Zakari Nikiéma, Timothée Kambou

**Affiliations:** ^1^Division of Urology, Souro Sanou University Teaching Hospital, Bobo-Dioulasso, Burkina Faso; ^2^Division of Urology, Yalgado Ouedraogo University Teaching Hospital, Ouagadougou, Burkina Faso; ^3^Division of Radiology and Imaging, Souro Sanou University Teaching Hospital, Bobo-Dioulasso, Burkina Faso

## Abstract

A 34-year-old man, trader, and married with four wives, otherwise healthy, without any remarkable medical history, was admitted in urology ward in emergency with superficial venous thrombosis of the penis known also as Penile Mondor's Disease (PMD), a rare nosologic entity of the penis associated with pain and an indurated dorsal cord of the root of the penis. The patient receives nonsteroidal anti-inflammatory drugs and coagulation and platelet aggregation inhibitors drugs. Healing and total recovery occur after eight weeks without any complications.

## 1. Introduction

Mondor's Disease (MD) is a rare nosologic entity, most often localized in the anterolateral thoracic wall and mammary region. It was first described by Henri Mondor in 1939 [1]. It is a superficial venous thrombosis occurring in a healthy vein but regress spontaneously. The thoracic and abdominal wall, abdominal wall, penis, upper arm, and other parts of the body may also be involved by the disease penile localization, known as Penile Mondor's Disease (PMD) which was first described by Braun Falco in 1955 [2] and then by Helm and Hodge in 1958 [3]. It is a rare and underreported benign genital condition and its clinical aspects are common and etiologies and risk factors are various. Nowadays, there are no standard strategy treatments of PMD and sometimes the management can combine several means. Usually, patient presents redness and swelling of the penis accompanied by painful palpable venous thrombosis of the penis. Pathogenesis is not well known but it is believed by most authors that it is due to pulling and torsion of the dorsal vein of the penis following microtrauma or during laborious sexual intercourse [4]. We present our first observation of this benign genital condition in our urology division in a young man who was admitted with this disease and was treated with success.

## 2. Case Presentation

**Figure 1:**
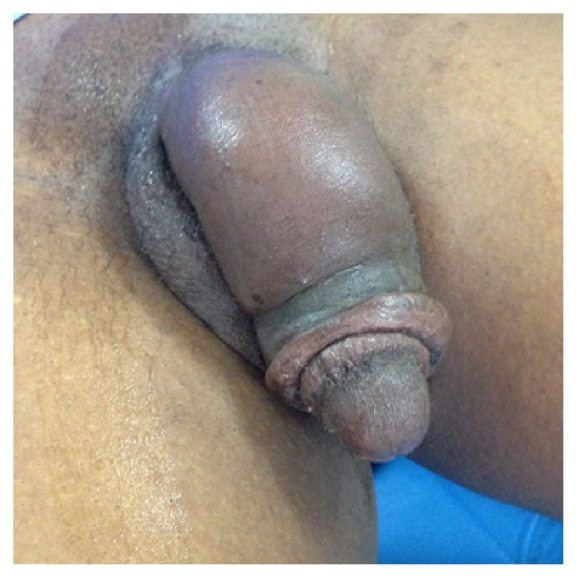
Generalized swelling of the penis.

A 34-year-old man, married with four women, trader, with no known past medical history, was admitted in emergency in the urology division at Souro Sanou University Teaching Hospital of Bobo-Dioulasso (Burkina Faso) for a painful swelling penis following laborious sexual intercourse. This symptomatology began nine (9) days later with progressive painful swelling at the balanopreputial ring just after sexual intercourse. The patient consulted previously in a private clinic and was given some medication without success, and then he is admitted in emergency in our urology ward because of the generalized involvement of the entire penis with painful oedema. Medical history does not find any recent trauma or use of any medication; no urinary tract symptoms were reported but recent intense sexual intercourse was noted. The patient reported that he is married to four women and regularly having vigorous sexual intercourse activities. Physical examination found an indurated subcutaneous filiform and painful cord, palpated at the dorsal root of the penis with soft swelling of the whole penis (Figure 1).

**Figure 2:**
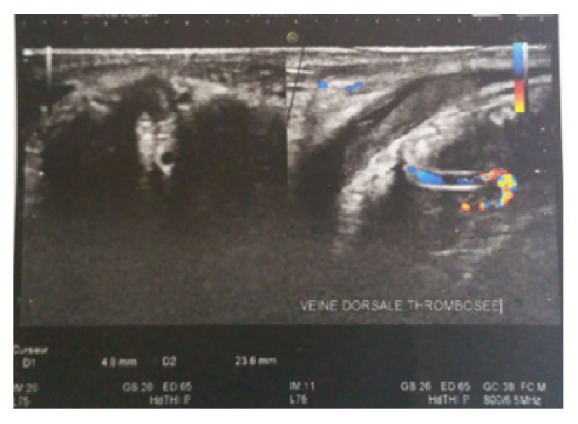
Doppler coupled with USS showing an intraluminal thrombosis (weight arrow) of the dorsal vein of the penis.

Examination of genito-urinary system was normal. Standard investigations (blood and urine) requested were normal, and blood cell count, urea, and creatinine were without anomalies. Urine analysis does not reveal any infection. Suspicion of penile fracture was made but anamnesis does not find a popping sound or hearing a cracking or losing an erection suddenly during sexual intercourse. Secondary, diagnosis of thrombosis of the superficial dorsal penile vein was made. An ultrasonography coupled with Doppler of the penis was requested and revealed a thrombosis of the superficial dorsal vein of the penis with the presence of the intravascular blood clot thrombosis (Figure 2).

**Figure 3:**
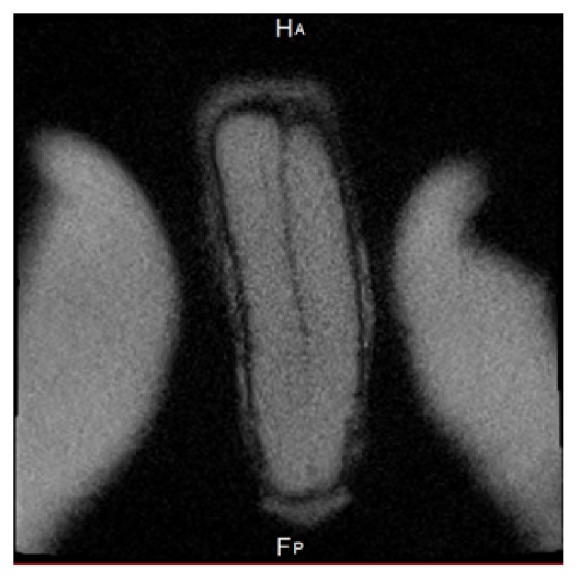
MRI of the penis showing the integrity of the cavernous bodies.

To be sure to excluded penile fracture, penile magnetic resonance imaging (MRI) was performed and do not reveal any traumatic lesion of cavernous and spongious bodies (Figure 3).

Conservative treatment was done with nonsteroidal anti-inflammatory drugs (NSAID) and acetyl salicylic acid stick (100mg/day) for thrombosis prevention by coagulation and platelet aggregation inhibition. The patient was informed about the necessity of sexual abstinence till disappearance of all symptoms. The evolution was marked by quick and favorable recovery with disappearance of pain in eight weeks, reduction of the swelling, and complete and total recovery of the penis. At six months of follow-up, no evidence of reoccurrence was observed.

## 3. Discussion

Penile Mondor's Disease (PMD) by superficial venous thrombosis of the dorsal vein of the penis described the first time by Braun Falco in 1955 is a benign genital condition [2]. It affects men with active sexual activities. Etiopathogenesis is not well known; many risks factors are being described. The main risk factor is excessive sexual activities, but also implicated are trauma, sexual abstinence, local infections as sexual transmitted disease, pelvic tumours, or use of vasoconstrictive drugs, bladder overdistension, use of vacuum erection device, and so on. Association of PMD with some urologic tumours as bladder cancer or prostate cancer has been reported and this pathology has been described as a first unusual manifestation of the metastatic pancreatic adenocarcinoma [5]. In our case, intense sexual activity was identified as a main risk factor. Our patient was married with four women, and he reported an intense sexual activity during the period when PMD have occurred. However Penile Mondor's Disease can occur without a clearly determined aetiology. Sickle cell disease could be also a risk factor as reported by some authors [6]. In fact, Nachmann and colleagues in their study on Penile Mondor's Disease argued that, during sickle cell disease crisis, PMD can occurr regarding possibility of the genesis of thrombus [7]. Diagnosis of Penile Mondor's Disease is usually made on clinical findings; thorough taking of the medical history and a correct physical examination are essential for diagnosing. A cord-like lesion with few centimeters in length can be easily recognized on inspection, and a hard induration can be palpated beneath the skin [3]. Sometime the lesion can extended cranially to the suprapubic region, and the vein will appear distended and erythematous like in the case of our patient. Laboratories tests and invasive diagnostic tool are not always mandatory to make the PMD diagnosis. The thrombosed superficial veins should first be detected with a gray-scale sonogram. Doppler coupled with ultrasound can be helpful in certain situations when the diagnosis is not evident. Doppler and US can be also used for follow-up, showing the recanalization of the endoluminal thrombosis vein after the total recovery of the patient. In our patient Doppler couple with Ultrasound, Magnetic Resonance Imaging of the penis was requested to ensure that there are no lesions in the cavernous and spongious bodies and thus the diagnosis of the superficial venous thrombosis was retained. Differential diagnosis of the painful swelling deforming penis may include penile fracture, Peyronie disease, which is a painful deviation of the shaft during erection, without palpation of the indurated dorsal cord like in PMD. The treatment of the superficial dorsal venous thrombosis of the penis varied from simple observation, conservative therapy, and anticoagulation drugs administration to radical treatment with surgery including thrombectomy and dorsal vein resection. General measures must be observed as sexual abstinence and treatment of underlying disease if present. In reported cases, where patient received no treatment, healing occurs after 6 or 8 weeks with recanalization of the thrombosed vein [7]. In acute state, proposed therapies are variable: antiplatelet aggregate agents, NSAID, heparin drugs for preventive and curative treatment for 15 days. In case where medical treatment was not effective or PMD which not responding to medical treatment, surgical thrombectomy of the superficial dorsal vein can be attempted [8]. Sasso F and colleagues distinguished three types of treatment according to the clinical stage. None of the proposed method has shown a significant reduction in the healing duration. [4]. For Walsh and colleagues, Penile Mondor's Disease is a benign condition with common presentation but an uncommon disease but its treatment is primarily symptomatic but may vary depending on possible underlying disease processes [9]. However antibiotics drug administration is associated when there is cellulitis or sexually transmitted diseases (STD) till remission of symptoms. Nazir SS and colleagues found that the local infiltration of anesthetics (bupivacaine 0.5%) around the thrombosed area considerably retrieved the pain [10]. Our patient received a nonsteroidal anti-inflammatory and antiplatelet aggregate drug and total resolution was achieved in 8 weeks.

## 4. Conclusion

Mondor's Disease localized in the penis is a rare and benign pathology that the pathogenesis is not yet clear and well known. The diagnosis is based on clinical findings and the investigations by Doppler coupled with ultrasound can contribute to the diagnosis. Conservative therapy with observation or NSAID anticoagulation agent administration and temporary sexual abstinence can occur healing with total recovery. Surgical treatment can be indicated when there is persistence of thrombosis after a long-term medical treatment.
